# Surface Wettability Modeling and Predicting via Artificial Neural Networks

**DOI:** 10.3390/ma18010191

**Published:** 2025-01-05

**Authors:** Katarzyna Peta

**Affiliations:** Institute of Mechanical Technology, Poznan University of Technology, 60-965 Poznan, Poland; katarzyna.peta@put.poznan.pl

**Keywords:** contact angle, wettability, roughness, artificial neural networks, manufacturing

## Abstract

Surface wettability, defined by the contact angle, describes the ability of a liquid to spread over, absorb or adhere to a solid surface. Surface wetting analysis is important in many applications, such as lubrication, heat transfer, painting and wherever liquids interact with solid surfaces. The behavior of liquids on surfaces depends mainly on the texture and chemical properties of the surface. Therefore, these studies show the possibility of modeling surface wettability by adjusting the parameters of the surface texturing process. The prediction of the contact angle describing the wettability of the surface was performed using artificial neural networks. In order to select the most effective prediction model, the activation functions of neurons, the number of hidden layers and the network training algorithms were changed. The neural network model presented in these studies is capable of predicting the contact angle with an efficiency defined by the coefficient of determination R^2^ between real and predicted contact angles of over 0.9.

## 1. Introduction

Surface wettability describes the behavior of a liquid on a solid surface [1]. There are three basic behaviors of liquids on surfaces: spreading, adhering and absorption [2]. The interaction of a liquid with a solid surface is mainly described by the contact angle [3]. It is assumed that for a contact angle greater than 90°, the surface is characterized by weak wettability and exhibits hydrophobic properties, in contrast to a contact angle smaller than 90°, when the surface is well wettable and described as hydrophilic [4]. Regardless of the mechanism of liquid–surface interaction, the contact angle is calculated in the same way as the angle formed between the line tangent to the liquid droplet and the line defining the surface of the solid [5]. Contact angle calculations are a fundamental indicator of the behavior of liquids on surfaces. Wettability studies are essential in the development of surfaces in contact with liquids in order to improve their anti-corrosion [6], self-cleaning [7], coating [8], lubrication [9] or heat transfer properties [10]. Wettability therefore affects a number of other functional properties of surfaces.

A surface close to perfectly smooth is characterized by a specific contact angle that depends on the chemical properties of the material and the energy state of the surface [11]. Therefore, the same surface texture, but different materials, may exhibit different wettability. Surface modification techniques are key to modeling surface wettability. A surface with hydrophobic properties can be modified to hydrophilic properties and vice versa [12]. Wettability modifications can be achieved by physical, chemical or mechanical effects on the surface [13]. Physical surface modification may include plasma activation [14] or ultraviolet radiation [15]. Chemical modifications mainly consist of introducing polar functional groups on the surface, including carboxyl, carbonyl or hydroxyl [16]. Mechanical modifications concern surface texturing by machining processes [17]. Another way to change the wettability of a surface is to apply coatings with specific wetting properties [18].

Surface wettability is mainly dependent on the type of material, surface free energy, surface microgeometry, temperature and type of liquid, mainly its viscosity, surface tension and polarity [19]. It is possible to adjust these factors to influence the expected wettability of the surface [20]. In many applications, some factors are constant, such as the temperature of the environment and the fluid, for example, in the analysis of the behavior of synovial fluid on implant surfaces. Some factors, mainly the parameters of the surface modification process and texturing, which can create different surface microgeometries, are relatively easily programmable [9]. Therefore, finding and understanding the relationships between texturing process parameters, surface topography characterization parameters [21] and contact angles are at the core of surface wettability modeling [12]. It also seems important to indicate the best scales of wetting phenomena observation, defined as the coefficient of determination between the contact angle and surface complexity in relation to scales [22]. This approach is the basis of multiscale analysis (scale-sensitive fractal analysis) [23].

Artificial neural networks are increasingly used for the prediction of material properties after the manufacturing process [24]. They owe their applicability to the ability to self-learn based on examples of training data sets, even if the relationships between the data are not fully clear. In this context, the use of artificial intelligence methods is superior to commonly used physical models [25]. Artificial neural networks are a computational system bioinspired by the functioning of the nervous system [26]. Neurons in the network form an input layer, to which input data are entered, hidden layers where calculations are performed and an output layer that outputs the result. The data processed by the neural network are activated by the activation function and transmitted to subsequent neurons [27]. The connections between neurons are assigned weights that can strengthen or weaken the influence of individual inputs on the network’s output. Biases, on the other hand, can shift activation to make the neural network more flexible [28]. The evaluation of the effectiveness of a neural network is mainly based on the calculation of the coefficient of determination between the real and predicted output data, as well as the calculation of the mean squared error [29]. Prediction of material properties using artificial neural networks is a current topic in the literature [30,31].

Previous research works on the prediction of surface topography characteristics after EDM machining include EDM parameter–surface roughness relationships. Paturi et al. studied the surface machinability in the EDM process using artificial neural networks. The aim of these works was to minimize the machining time while maintaining the assumed surface quality [32]. Khan et al. developed the modeling of the average roughness parameter Ra in the EDM process using neural networks. This research aimed to improve the economic factors of machining [33]. Tsai et al. presented the prediction of EDM surface finish using neural networks. The authors concluded that good accuracy in modeling the surface topography structure in EDM can be achieved despite the stochastic nature of this process [34].

The prediction of surface wettability is an interesting and important research topic as evidenced by studies described in the literature. Cho et al. developed a surface wettability prediction method based on artificial neural networks that recognize digital images of surface nanotextures created with oxygen plasma [35]. Choi et al. presented a neural network for predicting the wettability of a surface patterned with rectangular pillars, taking into account intermediate states of the droplet deposited on the surface according to the Wenzel and Cassie–Baxter models [36]. Baronti et al. described a neural network for predicting the functional response of laser-textured surfaces. The difference between the real and predictive contact angles was determined to be max. 10° [37]. Ibrahim et al. analyzed the CO_2_ wetting behavior in shale formations by using artificial neural networks to reduce the cost of experimental studies [38]. Kalliorinne et al. developed a neural network for the prediction of contact mechanics parameters in relation to roughness parameters. These studies explore the links between surface topographic characteristics and surface functional properties [39]. Huang et al. presented a neural network model for predicting surface wettability after laser processing. This model takes into account both surface texture and its chemical properties. The developed neural network model has good wettability prediction efficiency, but the authors recommended caution when applying this model to surfaces textured by other techniques [40].

Scientific work on supporting the EDM process in surface quality prediction mainly includes surface topography characteristics but does not go further into surface functional features. Neural networks are mainly used to solve one task. The novelty of this work is to extend this perception and find predictive models of both surface topography and surface wettability in EDM. Previous works describe the prediction of surface wettability after other processes, such as laser or plasma processing. Insufficient literature descriptions of surface wettability after EDM may result from the multitude of parameters shaping the surface in this process. It is therefore crucial to find those parameters that most affect the surface topography, as well as the contact angle.

The control system of most EDM machines enables estimation of the average surface roughness Ra in accordance with the VDI 3400 standard. Based on previous studies, it was observed that the Ra parameter is not the only one determined by the energy of electric discharges and has an impact on the surface wettability. The aim of these studies was therefore to identify the interdependencies between EDM parameters, surface topography and wettability in order to create a predictive model based on artificial neural networks.

Prediction of the wetting of aluminum alloy surfaces, described by the contact angle, has scientific and practical importance. From a scientific point of view, it allows us to learn about the relationships between the parameters of the manufacturing process, topographic characterization of the surface and wettability and also to verify the possibilities of predicting these properties using artificial intelligence tools. From a practical point of view, understanding these relationships is important wherever aluminum alloys cooperate with liquids, such as heat exchangers. Surface texture affects the efficiency of heat transfer, including the behavior of liquids in these systems.

The novelty of the research consists of the presentation of a multi-task artificial neural network for modeling surface wettability based on the modification of electrical discharge machining parameters. The neural network proposes the prediction of both the parameters of the topographical surface characterization and the functionally related property, in this case, wettability. The development of the neural network model was preceded by calculations of the correlations of process parameters, surface topography parameters and contact angles in order to include those data in the network that exhibit dependencies. Activation functions, the number of hidden layers and network training algorithms were compared to determine their impact on the predictive performance of the neural network. The performance of the model was assessed by the coefficients of determination between real and predicted data, as well as the mean square error. The aim of developing a neural network model for modeling surface wettability based on texturing process parameters is to improve the process of designing and controlling surface quality. Artificial intelligence methods are an additional tool supporting manufacturing, making it faster, cost-effective and more accurate.

## 2. Material and Methods

### 2.1. Wettability Model—General Assumptions

This study describes a generalized artificial neural network model, mainly indicating the most important inputs influencing the network output (surface wettability). Then, the implementation of an artificial neural network in a specific case study is presented. The research methodology presented in the example can be a guide for predicting the surface wettability of other materials textured via other surface texturing processes.

**Figure 1:**
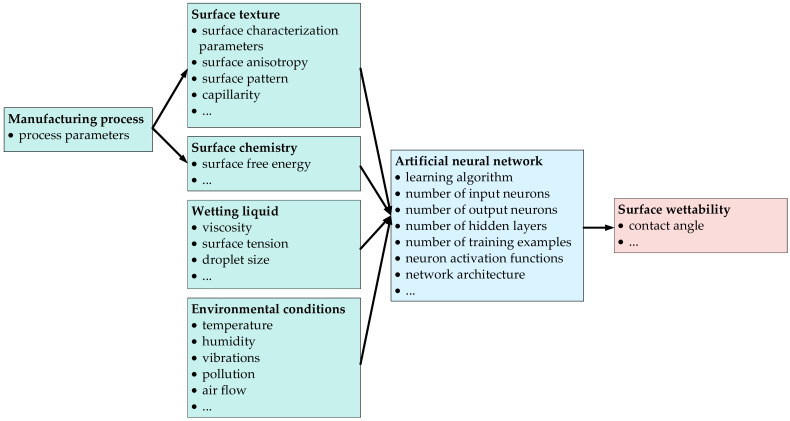
The schematic view of the model for predicting the wettability of the surface.

Modeling an artificial neural network begins with identifying the inputs and outputs. The output of the neural network, in this context, wettability, is influenced by surface texture, surface chemistry, wetting fluid properties and environmental conditions. However, the texture and chemical properties of the surface are determined by the manufacturing process. The data on which the neural network calculations are based should be relevant, specific and achievable to measure or determine. Thus, in more detail, it could be stated that the parameters of the manufacturing process generate a surface with specific parameters of surface topographic texture and surface energy. Adding to this the properties of the wetting liquid, such as viscosity, surface tension and environmental conditions, such as temperature and humidity, the contact angle can be predicted. The general model of the artificial neural network, taking into account the most important input data and the output data of the network, is shown in Figure 1.

### 2.2. Surface Manufacturing

The experimental part describes the case of predicting the wettability of the 6060 aluminum alloy surface based on the electrical discharge machining (EDM) parameters, indirectly determining the topographic surface characterization parameters and the surface free energy. This task is a continuation of the publication [12], which discussed the influence of EDM parameters on the texture and energy state of the surface, affecting the functional surface feature and wetting, at different scales of observation. The results of the studies presented there showed correlations between the energy of electrical discharges, selected topographic surface characterization parameters and contact angles. The occurrence of strong determination coefficients R^2^ > 0.8 is a prerequisite for the possibility of modeling and predicting surface wettability.

Surfaces of 6060 aluminum alloy in T4 condition (extruded and then cooled) with dimensions of 40 mm × 40 mm × 5 mm were electro-discharge machined on an Agie Charmilles Form 20, GF Machining Solutions (Biel/Bienne, Switzerland). A copper electrode with dimensions of 41 × 41 mm was used in the process so that the electrode movements during the process took place only on the z axis. The dielectric liquid was distilled water.

The EDM parameters were based on the internal machine control system, which allowed the selection of the expected average surface roughness resulting from the VDI classes (VDI 3400 standard). The selection of the average roughness parameter allows the automatic selection of EDM parameters. The key is that the system does not allow for indicating all surface characterization parameters or functional features of the surface. Therefore, this was considered an area for research in these studies.

**Figure 2:**
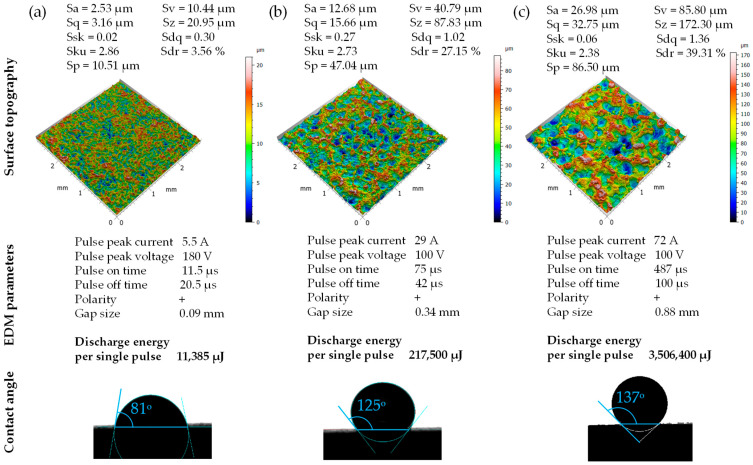
Views of three examples of 3D images of the surface after EDM with discharge energies per single pulse: (**a**) 11,385 µJ, (**b**) 217,500 µJ, (**c**) 3,506,400 µJ.

The parameters of electro-discharge machining that are important from the point of view of the created surface topography are current, voltage, pulse duration, pulse pause time, polarity and gap size. Figure 2 shows images of three example surfaces obtained after the EDM process, along with the EDM parameters used to create them. One of the factors determining the surface texture after EDM is the discharge energy calculated as the product of current, voltage and pulse duration.

### 2.3. Measurement Methods

This texturing method allows us to obtain isotropic surfaces that are characterized by similar wettability in all directions. However, these surfaces are characterized by a certain irregularity of peaks and valleys, which result from the EDM process itself. Therefore, it is important to perform several repetitions of surface wettability measurements in different positions. In this study, 7 measurements of surface topography parameters and the same number of contact angle measurements were performed for each surface. Surface topography measurements were performed on a Bruker Alicona 3D optical microscope (Bruker, Billerica, MA, USA), and contact angle measurements were performed on an OCA 15 Pro goniometer from DataPhysics (Filderstadt, Germany). The parameters of these measurements are given in Table 1. Surface topographies were processed in MountainsMap version 9 software (DigitalSurf, France), and surface wetting droplets were processed in SCA 20 software (DataPhysics).

### 2.4. Artificial Neural Network Parameters

**Figure 3:**
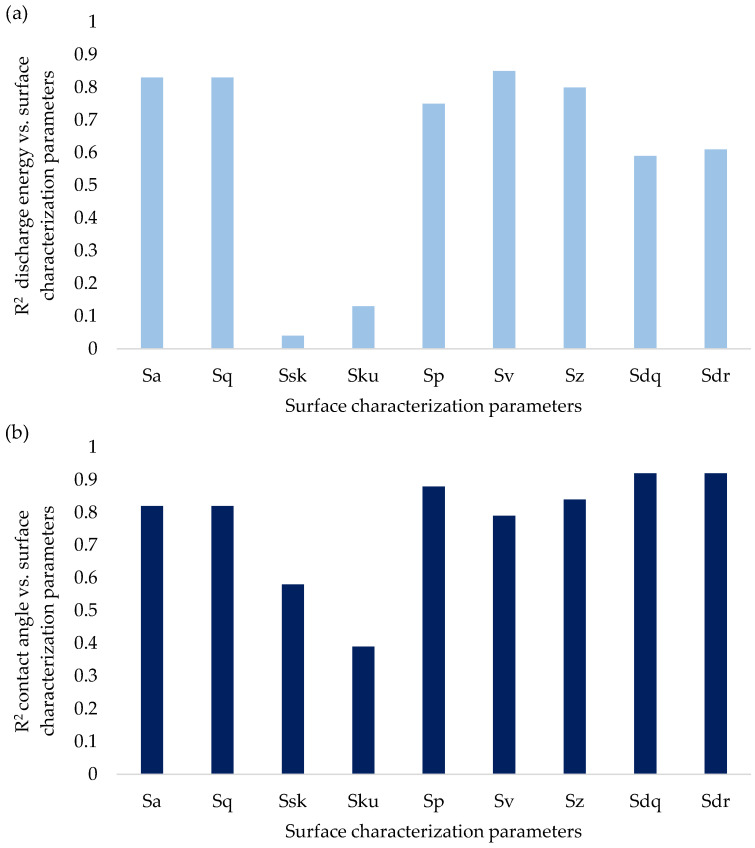
Determination of coefficients R^2^ between (**a**) the discharge energy in the EDM process and (**b**) the surface topographic characterization parameters and contact angles.

The selection of input data for the neural network was based on the calculation of the coefficients of determination R^2^ between the EDM process parameters that create the discharge energy and the parameters of the surface topography characterization specified in the ISO 25178-2 standard (Figure 3). The next step included the calculation of the coefficients of determination R^2^ between the parameters of the surface topography characterization and the contact angle (Figure 3). These correlation analyses allowed us to indicate which parameters are dependent on each other and can be effectively processed by the neural network.

Most of the surface characterization parameters from the height and hybrid groups correlate well with the EDM parameters and contact angles. Only the parameters Ssk and Sku do not show sufficient correlations. Therefore, modeling the symmetry of the surface height (Ssk) and also the peaks and valleys that deviate from the mean plane (Sku) is not effective in EDM. Particularly good results R^2^ > 0.8 for both discharge energy and contact angles correlating with the surface topographic characterization parameters are achieved by the arithmetical mean roughness (Sa), root mean square height (Sq), maximum pit height (Sv) and maximum height (Sz). It can therefore be assumed that there is a high chance that these parameters will effectively contribute to modeling the wettability of EDM-textured surfaces.

An artificial neural network model for modeling the wettability of electro-discharge-textured surfaces considered in this case was developed based on the EDM parameters of current, voltage and pulse duration, as network input data, and based on them, four parameters of surface topographic characterization that best correlate with discharge energy and contact angle, i.e., Sa, Sq, Sv and Sz, were predicted. A neural network output was then generated, known as the contact angle, which determines the surface wettability. The model of this neural network does not take into account the properties of the liquid wetting the surface and the environmental conditions. It was assumed that these parameters are constant for each analyzed surface, so their influence on the generated result would be negligible.

All data constituting training cases for the neural network have numerical values, the normalization of which is the basis for minimizing redundancy and inconsistent dependence between data. Therefore, all numerical data (X_normalized) were scaled to values from the range 0–1 according to a linear transformation and min-max function. The minimum value (min (X)) is subtracted from the scaled value, and the result of this calculation is divided by the difference between the maximum (max (X)) and minimum value:

(1) $$X\_normalized=\frac{X-min(X)}{\max\left(X\right)-min(X)}.$$

In order to develop the most effective neural network model, 250 models with different parameters and architectures were compared. The compared neural network models included combinations of the following parameters and architectures:

- Neural network learning algorithms (Broyden–Fletcher–Goldfarb–Shanno, steepest gradient, radial basis function teaching, scaled conjugate gradient);
- Neural network architectures (multilayer perceptron, radial basis function network);
- Neuron activation functions (linear, exponential, sigmoidal, sine, hyperbolic tangent, gaussian);
- Number of hidden layers with hidden neurons (1, 2, …, 7).

The criterion for evaluating the network is the coefficient of determination R^2^ between predicted data and real data. A coefficient of determination R^2^ closer to 1 means a better match between predicted and real data and thus a greater efficiency of the neural network. The neural networks were also evaluated in terms of prediction error calculated from the sum of squares (SOS) equation, in which the squares of the differences between the experimental and predictive results are summed:

(2) $$Sum\_of\_squares=\sum_{i=1}^{n}{\left(y_{i}-y_{i}^{*}\right)}^{2}.$$

## 3. Results and Discussion

**Figure 4:**
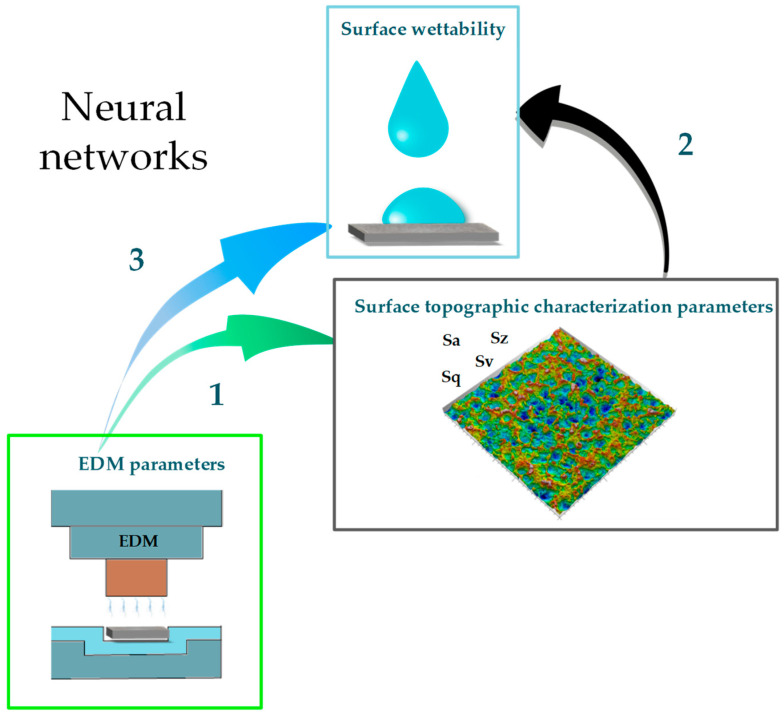
Schematic view of prediction tasks performed by artificial neural networks. 1—prediction of surface topography parameters based on EDM parameters, 2—prediction of surface wettability based on surface topography parameters, 3—prediction of surface wettability based on EDM parameters.

The prediction of wettability was carried out in three stages (Figure 4). Therefore, an artificial neural network was modeled to indicate the parameters of the topographical surface characterization based on the EDM parameters (1). This allows us to indicate the possibility of creating an irregular texture of the eroded surface with a specific crater size by changing the discharge energy parameters. Confirmation of the relationship between the manufacturing process and the created texture allows us to proceed further and design an artificial neural network to predict surface wettability dependent on the topographical surface characterization parameters (2). Identification of the relationships between the process, surface and functionality allows us to conclude on the justification of developing an artificial neural network to determine the surface wettability directly based on the EDM parameters (3).

**Figure 5:**
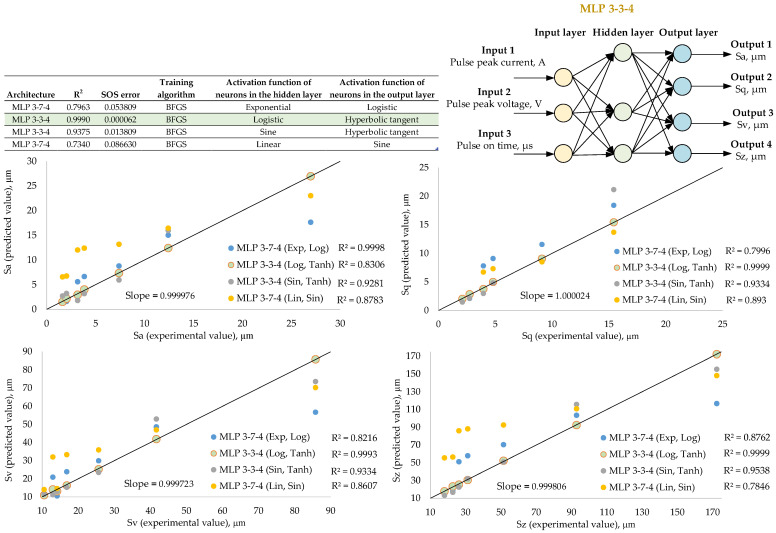
Results of a neural network for predicting surface topography parameters based on EDM parameters. The green background color indicates the parameters of the neural network with the best coefficient of determination R^2^ between the experimental and predicted results.

**Figure 6:**
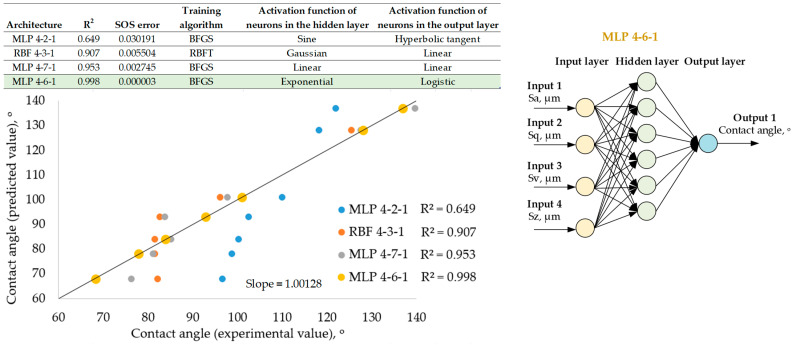
Results of a neural network for predicting surface wettability based on surface topography parameters. The green background color indicates the parameters of the neural network with the best coeffi-cient of determination R^2^ between the experimental and predicted results.

**Figure 7:**
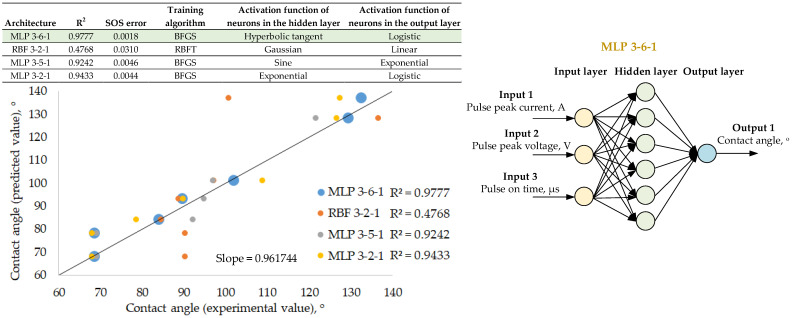
Results of a neural network for predicting surface wettability based on EDM parameters. The green background color indicates the parameters of the neural network with the best coeffi-cient of determination R^2^ between the experimental and predicted results.

Figure 5, Figure 6 and Figure 7 present artificial neural network models with the highest prediction efficiency calculated based on the determination coefficient R^2^ between the network input and output parameters. The relationship between the highest determination coefficient and the lowest sum-of-squares error is noticeable. Each of the proposed networks is described by its architecture, coefficient of determination and sum-of-squares error but also by the network training algorithm and the activation functions of hidden and output neurons. Regression graphs between experimental and predictive values are presented for four selected network models from 250 variants of different networks. The experimental results were obtained in the laboratory by carrying out surface topography and wetting measurements on previously EDM-textured surfaces. The predictive results are based solely on neural network simulations. The purpose of verifying the network efficiency is to assess the differences between the experimental and predictive results. These studies also included the selection of the most effective neural network model along with the presentation of its architecture, consisting of the network type (multilayer perceptron (MLP) or radial basis function (RBF) networks) as well as the number of input, hidden and output neurons.

The results of the prediction of surface topographic characterization parameters based on electrical discharge machining parameters are presented in Figure 5. The most effective network has the MLP 3-3-4 architecture. It is a multilayer perceptron with three input neurons to which the current, voltage and pulse on time parameters per single impulse are entered (EDM parameters). The next calculations are performed in three hidden neurons with a logistic activation function. Later, the output of the network is determined and activated by the hyperbolic tangent function. The output consists of four surface topography parameters Sa, Sq, Sv and Sz. The MLP architecture defines a multilayer perceptron in structure, which has one input layer, one or more hidden layers and one output layer. The training of this type of network is mainly based on the use of the backward error method. In MLP networks, there is no internal feedback. This network has a high coefficient of determination between the experimental and predictive results R^2^ > 0.95. The networks with different architectures generate greater discrepancies between these results according to the prediction efficiency. The next network generating results higher than R^2^ > 0.9 is the 3-3-4 MLP network, with hidden neurons activated by the sine function and output neurons activated by the hyperbolic tangent function.

Figure 6 presents the results of surface wettability prediction based on the surface topographic characterization parameters. The network input consists of four topographic surface characterization parameters Sa, Sq, Sv and Sz, and the network output is the contact angle. The best efficiency is characterized by the MLP 4-6-1 architecture, whose hidden neurons are activated by the exponential function, and the output neuron is activated by the logistic function. Such a network structure allows us to obtain the coefficient of determination R^2^ > 0.95. Two more network models were detected that allowed us to obtain slightly worse results but at the level of the determination coefficient R^2^ > 0.9, and these are MLP 4-7-1 and MLP 4-3-1.

The relationships between the surface topography parameters and the EDM parameters, as well as the wetting parameter, enable effective prediction. It is therefore justified to conclude that it is possible to model the surface wettability by appropriately adjusting the EDM process parameters. Figure 7 presents the results of the neural network for the prediction of the contact angle based on the EDM parameters. The highest coefficient of determination R^2^ > 0.95 and the smallest sum-of-squares error of 0.0018 were obtained for the MLP 3-6-1 architecture network with hidden neurons activated by the hyperbolic tangent function and an output neuron activated by the logistic function. The MLP 3-5-1 and MLP 3-2-1 networks obtained lower values of the coefficient of determination but still higher than R^2^ > 0.9. A common feature of all MLP networks with a coefficient of determination between the experimental and predictive results is the effectiveness of the BFGS (Broyden–Fletcher–Goldfarb–Shanno) network training algorithm.

It is usually assumed that adding additional layers of hidden neurons allows for deeper learning of the neural network and obtaining more accurate results. The limitation of this approach is primarily the increase in calculation time. The optimum is considered to be three layers of hidden neurons [41], but this selection depends mainly on the expectations in the time/accuracy relationship of the prediction task.

## 4. Conclusions

These studies aimed to show the possibility of modeling and predicting surface microgeometry and wettability based on electrical discharge machining parameters. EDM parameters that generate electrical discharge energy affect the size of surface craters, which determine the contact angle.

Artificial neural network models for predicting surface characterization parameters and wettability are characterized by high efficiency described by the coefficient of determination between experimental and predictive results R^2^ > 0.95. The performance of a network is influenced by the network training algorithm, activation functions and the number of hidden neuron layers.

Modeling and predicting surface wettability is a valuable method at the stage of surface design and specific functional features but also for verifying their quality. Artificial neural networks can be an additional tool that improves efficiency and reduces the time of engineering analyses. Further work may include verification of the effectiveness of the developed artificial neural network models for other materials and surface texturing techniques.

## Figures and Tables

**Table 1:** Surface topography and contact angle measurement parameters.

Surface Topography Measurement Parameters		Contact Angle Measurement Parameters	
Magnification	20×	Liquid type	distilled water
Lateral dimensions	2800 × 2800 µm	Droplet size	2 µL
Lateral sampling interval	0.49 µm	Droplet dosing speed	1 µL/s
Lateral resolution	2.9 µm		
Vertical resolution	<50 nm		

## Data Availability

Data are contained within the article.

## References

[1] Jafari M., Jung J. (2017). Direct measurement of static and dynamic contact angles using a random micromodel considering geological CO_2_ sequestration. Sustainability.

[2] Szymczyk K., Zdziennicka A., Krawczyk J., Jańczuk B. (2012). Wettability, adhesion, adsorption and interface tension in the polymer/surfactant aqueous solution system. I. Critical surface tension of polymer wetting and its surface tension. Colloids Surf. A Physicochem. Eng. Asp..

[3] Rbihi S., Aboulouard A., Laallam L., Jouaiti A. (2020). Contact Angle Measurements of Cellulose based Thin Film composites: Wettability, surface free energy and surface hardness. Surf. Interfaces.

[4] Yoshimitsu Z., Nakajima A., Watanabe T., Hashimoto K. (2002). Effects of surface structure on the hydrophobicity and sliding behavior of water droplets. Langmuir.

[5] Young T.J., Jackson J., Roy S., Ceylan H., Sundararajan S. (2017). Tribological behavior and wettability of spray-coated superhydrophobic coatings on aluminum. Wear.

[6] Chen Z., Wu C., Zhou H., Zhang G., Yan H. (2022). A high-efficiency preparation method of super wear-resistant superhydrophobic surface with hierarchical structure using wire electrical discharge machining. Surf. Coat. Technol..

[7] Shen D., Ming W., Ren X., Xie Z., Liu X. (2021). Progress in Non-Traditional Processing for Fabricating Superhydrophobic Surfaces. Micromachines.

[8] Pogorzelski S., Boniewicz-Szmyt K., Grzegorczyk M., Rochowski P. (2022). Wettability of Metal Surfaces Affected by Paint Layer Covering. Materials.

[9] Peta K., Bartkowiak T., Rybicki M., Galek P., Mendak M., Wieczorowski M., Brown C.A. (2024). Scale-dependent wetting behavior of bioinspired lubricants on electrical discharge machined Ti6Al4V surfaces. Tribol. Int..

[10] Ye X.M., Zhang N.K., Cheng R., Li C.X. (2022). Effect of Contact Angle Hysteresis on Evaporation Dynamics of a Sessile Drop on a Heated Surface. J. Appl. Fluid Mech..

[11] Stammitti-Scarpone A., Acosta E.J. (2019). Solid-liquid-liquid wettability and its prediction with surface free energy models. Adv. Colloid Interface Sci..

[12] Peta K. (2024). Multiscale Wettability of Microtextured Irregular Surfaces. Materials.

[13] Cavalu S., Antoniac I.V., Mohan A., Bodog F., Doicin C., Mates I., Ulmeanu M., Murzac R., Semenescu A. (2020). Nanoparticles and nanostructured surface fabrication for innovative cranial and maxillofacial surgery. Materials.

[14] Kim H.Y., Kang B.H. (2003). Effects of hydrophilic surface treatment on evaporation heat transfer at the outside wall of horizontal tubes. Appl. Therm. Eng..

[15] Yin H., Moghaddam M.S., Tuominen M., Dėdinaitė A., Wålinder M., Swerin A. (2022). Wettability performance and physicochemical properties of UV exposed superhydrophobized birch wood. Appl. Surf. Sci..

[16] Kim S., Marcano M.C., Becker U. (2021). Effects of Hydroxyl and Carboxyl Functional Groups on Calcite Surface Wettability Using Atomic Force Microscopy and Density Functional Theory. ACS Earth Sp. Chem..

[17] Dong J., Liu Y., Pacella M. (2024). Surface Texturing and Wettability Modification by Nanosecond Pulse Laser Ablation of Stainless Steels. Coatings.

[18] Woźniak A., Adamiak M., Chladek G., Bonek M., Walke W., Bialas O. (2020). The influence of hybrid surface modification on the selected properties of cp titanium grade ii manufactured by selective laser melting. Materials.

[19] Leena K., Athira K.K., Bhuvaneswari S., Suraj S., Rao V.L. (2016). Effect of surface pre-treatment on surface characteristics and adhesive bond strength of aluminium alloy. Int. J. Adhes. Adhes..

[20] Skondras-Giousios D., Karmiris-Obratański P., Jarosz M., Markopoulos A.P. (2024). Investigation of the Influence of Machining Parameters and Surface Roughness on the Wettability of the Al6082 Surfaces Produced with WEDM. Materials.

[21] (2012). Geometrical Product Specifications (GPS)—Surface Texture: Areal—Part, 2.

[22] Brown C.A. (2021). Surface Metrology Principles for Snow and Ice Friction Studies. Front. Mech. Eng..

[23] Brown C.A., Hansen H.N., Jiang X.J., Blateyron F., Berglund J., Senin N., Bartkowiak T., Dixon B., Le Goïc G., Quinsat Y. (2018). Multiscale analyses and characterizations of surface topographies. CIRP Ann..

[24] Peta K., Zurek J. Prediction of air leakage in heat exchangers for automotive applications using artificial neural networks. Proceedings of the 2018 9th IEEE Annual Ubiquitous Computing, Electronics & Mobile Communication Conference (UEMCON).

[25] Kleinmann S., Hertel J., Stetter R. (2012). Feasibility of neural networks for self-learning diagnosis systems. IFAC Proc. Vol..

[26] Hong J., Sun X., Peng J., Fu Q. (2024). A Bio-Inspired Probabilistic Neural Network Model for Noise-Resistant Collision Perception. Biomimetics.

[27] Farea A., Yli-Harja O., Emmert-Streib F. (2024). Understanding Physics-Informed Neural Networks: Techniques, Applications, Trends, and Challenges. AI.

[28] Rejfek L., Nguyen T.N., Chmelar P., Beran L., Tran P.T. (2019). Neural networks application for processing of the data from the FMICW radars. Symmetry.

[29] Zou B., Chibawe M., Hu B., Deng Y. (2023). A comparative analysis of artificial neural network predictive and multiple linear regression models for ground settlement during tunnel construction. Arch. Civ. Eng..

[30] Honysz R. (2021). Modeling the chemical composition of ferritic stainless steels with the use of artificial neural networks. Metals.

[31] Churyumov A.Y., Kazakova A.A. (2023). Prediction of True Stress at Hot Deformation of High Manganese Steel by Artificial Neural Network Modeling. Materials.

[32] Paturi U.M.R., Cheruku S., Salike S., Pasunuri V.P.K., Reddy N.S. (2022). Estimation of machinability performance in wire-EDM on titanium alloy using neural networks. Mater. Manuf. Process..

[33] Rahman Khan M.A., Rahman M.M., Kadirgama K. (2014). Neural network modeling and analysis for surface characteristics in electrical discharge machining. Procedia Eng..

[34] Tsai K.M., Wang P.J. (2001). Predictions on surface finish in electrical discharge machining based upon neural network models. Int. J. Mach. Tools Manuf..

[35] Cho Y., Kim S., Park C.H. (2022). Surface Wettability Prediction Using Image Analysis and an Artificial Neural Network. Langmuir.

[36] Choi S., Byun K., Jang J. (2021). Neural network modelling of the wettability of a surface grooved with the nanoscale pillars. Chem. Phys. Lett..

[37] Baronti L., Michalek A., Castellani M., Penchev P., See T.L., Dimov S. (2022). Artificial neural network tools for predicting the functional response of ultrafast laser textured/structured surfaces. Int. J. Adv. Manuf. Technol..

[38] Ibrahim A.F., Elkatatny S. (2023). Data-driven models to predict shale wettability for CO_2_ sequestration applications. Sci. Rep..

[39] Kalliorinne K., Larsson R., Pérez-Ràfols F., Liwicki M., Almqvist A. (2021). Artificial Neural Network Architecture for Prediction of Contact Mechanical Response. Front. Mech. Eng..

[40] Huang W., Samanta A., Chen Y., Baek S., Shaw S.K., Ding H. (2021). Machine learning model for understanding laser superhydrophobic surface functionalization. J. Manuf. Process..

[41] Uzair M., Jamil N. Effects of Hidden Layers on the Efficiency of Neural networks. Proceedings of the 2020 IEEE 23rd International Multitopic Conference (INMIC).

